# Effect of Magnetized Water on the Mechanical and Durability Properties of Concrete Block Pavers

**DOI:** 10.3390/ma11091647

**Published:** 2018-09-07

**Authors:** Saeid Ghorbani, Mostafa Gholizadeh, Jorge de Brito

**Affiliations:** 1 Civil Engineering Department, Faculty of Engineering, Ferdowsi University of Mashhad, Mashhad 91775-1111, Iran; Saeid.ghorbani@mail.um.ac.ir; 2 Chemistry Department, Faculty of Science, Ferdowsi University of Mashhad, Mashhad 91775-1436, Iran; m_gholizadeh@um.ac.ir; 3 Department of Civil Engineering, Architecture and Georresources, Instituto Superior Técnico, Universidade de Lisboa, 1049-001 Lisbon, Portugal

**Keywords:** concrete block pavers, magnetized water, blended cement, mechanical and durability properties, permanent magnetic field

## Abstract

In this research, the effect of magnetized water on the mechanical and durability behavior of concrete block pavers was investigated. For this purpose, a total of five mixes were prepared with water that passed through a permanent magnetic field 10, 20, 40, and 80 times at a constant speed of 2.25 m/s. Compressive strength, splitting tensile strength, flexural strength, resistance to sulfuric acid attack, water absorption tests, and Scanning Electron Microscopy (SEM) analyses were conducted. The compressive strength, splitting tensile strength, and flexural strength test results showed a significant positive effect of using magnetized water. The remaining tests also revealed that using magnetized water increases the resistance of concrete block pavers to sulfuric acid attack and decreases their water absorption.

## 1. Introduction

Concrete block pavers are solid blocks that are closely placed to form a pavement surface. They are placed on a thin layer of sand or filler to make a sub-base. A light-duty block pavement consists of five independent layers as follows: (a) surface course of concrete blocks, (b) laying course, (c) road base, (d) sub-base, and (e) subgrade [1]. The joints between concrete blocks are filled with suitable fine materials. Concrete block pavers are manufactured under controlled conditions by compacting dry concrete in a plastic or steel mold. Dry concrete is made of Portland cement, water, and fine and coarse aggregates, and has a low paste content compared with ordinary concrete [2]. In order to achieve a strong and durable paving surface, the dry concrete is subjected to vibration and pressure.

Concrete block pavers fall into two categories: interlocking pavers and architectural pavers. Interlocking pavers were invented by the Dutch after World War II in the early 1950s as a replacement for brick pavers, which were their traditional paving material, when they were in short supply due to post-war building construction. Architectural pavers provide more aesthetic alternatives and are widely used in architectural applications. Through these years, concrete block pavers have become an attractive engineering and economical alternative to both flexible and rigid pavements [3]. Concrete block pavers’ low maintenance and cost, strength, durability, and aesthetically pleasing surfaces compared to other pavements have made them more attractive for a variety of applications such as street roads, small and medium market roads, parking areas, pedestrian walks, and traffic intersections. However, concrete block pavements are widely used in built-up areas where a speed limit below 40 miles/h is normally imposed [4]. By using concrete block pavers, pavement materials are not wasted, and jack hammers or heavy equipment are not required [5].

Water chemistry (mineralogy, types of ions present, total dissolved solids, pH, etc.) is a key factor in concrete production that affects the mechanical properties of concrete, namely its compressive strength, flexural strength, water absorption, workability, and durability [6,7,8,9]. However, few investigations had been conducted on the effect of magnetized water on the mechanical properties of concrete mixes.

**Figure 1:**
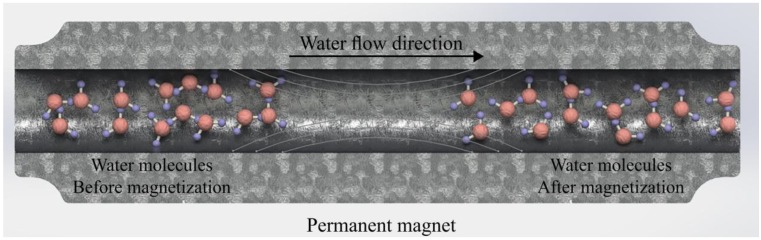
Effect of magnetic field on water molecule clusters.

Magnetized water has different mechanical, electromagnetic, and thermodynamic properties compared to regular tap water [8]. Due to these specific properties, the use of magnetized water has been increasing in different applications such as in industrial, environmental, medical, and agricultural fields due to the development of magnetic devices. The magnetization procedure of water is a simple method without using extra energy when a permanent magnet is used. The permanent magnet can be installed on a previously established water tube system, resulting in no further energy requirements for water magnetization [10]. Magnetized water is obtained when water passes through a permanent magnetic field with a constant speed. When that happens, some definite changes occur in its molecular characteristics. The molecules of regular tap water are not separated from each other due to the existence of hydrogen bonds. They tend to attach to each other, forming clusters. As regular tap water passes through a permanent magnetic field, the size of these clusters and the number of grouped molecules decreases [10,11,12]. Consequently, the activity of the water molecules increases. Toledo et al. [11] reported that the magnetic fields weakened the intraclusters’ hydrogen bonds, breaking the larger clusters, forming smaller clusters with stronger intracluster hydrogen bonds. The effect of the magnetic field in enhancing the hydrogen bonding was confirmed by Inaba et al. [13] and Cai et al. [14]. The effect of the magnetic field on water molecules is schematically shown in Figure 1.

Magnetized water has a lower surface tension than regular tap water, which is measured using a device called a tensiometer [15]. This lower surface tension affects the hydration and hardening process of cement particles. As the water and cement mix, the hydration process of cement will first take place on the surface of the cement particles. Consequently, a thin layer of hydration products is formed on the cement particles, which hinders the further hydration of the cement particles [16]. The effect of this action will be to hinder the development of the mechanical strength of the concrete. Passing the water through a permanent magnetic field prevents the accumulation of cement particles and also causes the water molecules to penetrate more easily into the cement particles, further developing the hydration process of the concrete mix [17]. Consequently, the mechanical properties of the concrete mix will improve [6,15,16,17,18,19]. It has been reported that the magnetization effect on the regular tap water can remain for hours or days after the magnetization of regular tap water [10,20]. Therefore, magnetic water affects the first days of the cement hydration process.

Few investigations have been recently conducted on the effect of magnetized water on the mechanical properties of concrete mixes. Su and Wu [18] studied the effect of magnetic field-treated water on mortar and concrete containing fly ash. The results showed that using magnetized water instead of regular tap water can improve the compressive strength and the flow ability of mortar specimens containing fly ash compared to mortars prepared with regular tap water. In another study, Su et al. [16] reported that using magnetized water instead of regular tap water can also improve the compressive strength of mortar specimens containing granulated blast-furnace slag. However, the rate of increase in compressive strength is varied, and depends on the strength of the permanent magnetic field. Bharath et al. [21] showed that the use of magnetized water enhanced the workability of concrete mixes containing copper slag as a partial replacement of cement by about 50%. Similarly, Gholhaki et al. [17] reported that the use of magnetized water instead of regular tap water can improve the flowability and viscosity of self-compacting concrete (SCC). Ghods [22] also showed that the use of magnetized water can improve the early-age compressive and tensile strengths of SCC mixes incorporating nano silicate. Other researchers also reported that using magnetized water instead of regular tap water in concrete production increases the workability and strength of the concrete mix without adding more water or any other materials such as plasticizer [19,23]. Faris et al. [19] found that the molecules of magnetized water have a lower surface tension than regular tap water, which leads to a higher activity of the cement particles in the mix. Ahmed [15] investigated the behavior of magnetic concrete incorporating Egyptian nano alumina, and concluded that a significant positive effect on the characteristics of concrete mix was obtained. Siva et al. [24] reported that the use of magnetized water in concrete production can improve the tensile and flexural strength of concrete mixes. Wei et al. [6] showed that the use of magnetized water in concrete production improved the early-age shrinkage cracking resistance of concrete mix compared to the specimens prepared with regular tap water. Using magnetized water instead of regular tap water to produce concrete mixes also decreases the amount of cement that is used to produce concrete mixes by about 5%, and it can also prevent the concrete mix from freezing [25,26,27].

Since no research has been conducted on the effect of magnetized water on the mechanical and durability properties of concrete block pavers, the aim of this investigation is to evaluate that effect, namely on the compressive strength, splitting tensile strength, flexural strength, resistance to sulfuric acid, and water absorption.

## 2. Materials and Sample Preparation

### 2.1. Materials

The materials that were used in this study to produce concrete block pavers include cement, water, fine, and coarse aggregates were locally manufactured in Iran. The cement used in this study was type II Portland cement manufactured by the Mashhad Cement Company (Mashhad, Iran) and conforming to American Society for Testing and Materials (ASTM) C150. The chemical composition of the cement is shown in Table 1.

**Figure 2:**
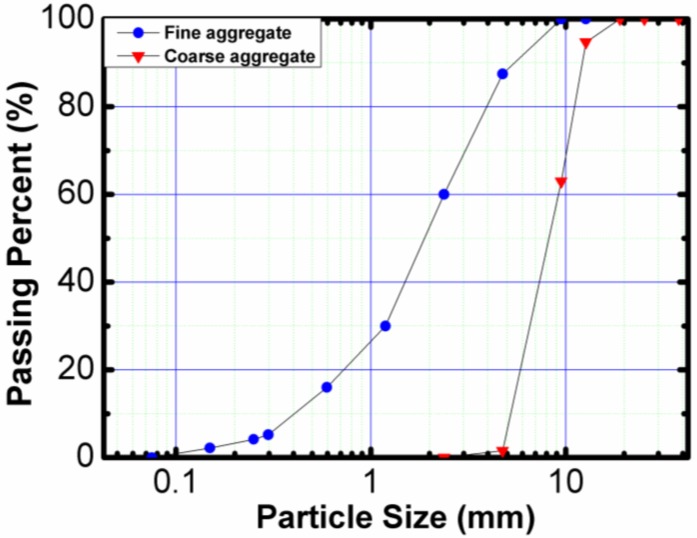
Particle size distribution of fine and coarse aggregates.

The coarse aggregates used in this study were crushed limestone, and the fine aggregates were river sands acquired locally. The physical properties of the fine and coarse aggregates and their particle size distribution are shown in Table 2 and Figure 2, respectively.

**Figure 3:**
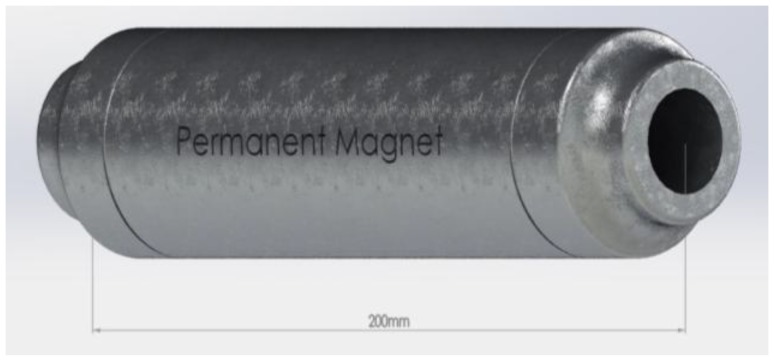
Permanent magnet used in this study.

Regular tap water was used for mixing. In order to magnetize regular tap water, a permanent magnet with a magnetic strength of 0.65 T was used. The magnet has a length of 200 mm, an internal diameter of 32 mm, and an external diameter of 55 mm. The permanent magnet used in this study is schematically shown in Figure 3.

**Figure 4:**
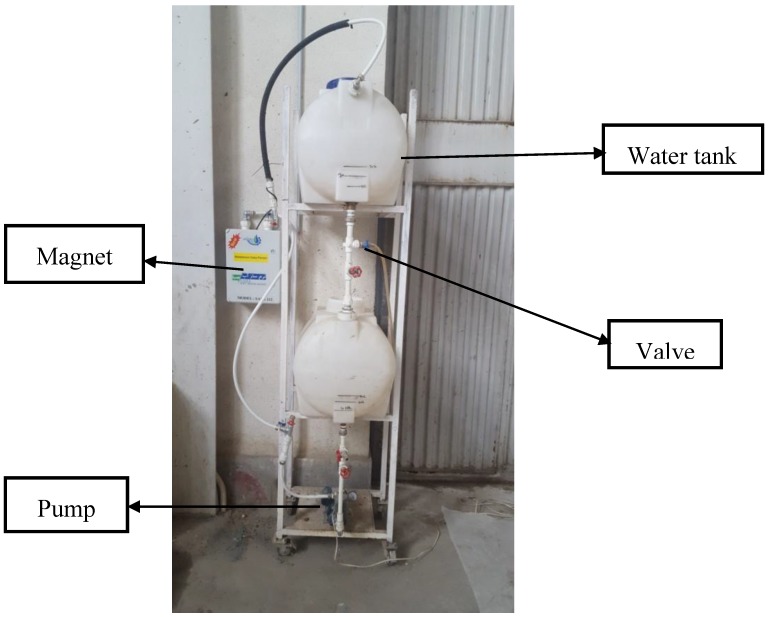
Device to produce magnetized water.

In order to prepare the magnetized water, regular tap water was circulated through the permanent magnet at a constant speed and water flow of 2.25 m/s 10, 20, 40, and 80 times. One end of the magnet was connected to a pump, and the other end was connected to a water tank, as shown in Figure 4.

### 2.2. Experimental Design

The experimental program intends to investigate the effect of magnetized water on the compressive strength, splitting tensile strength, flexural strength, resistance to sulfuric acid, and water absorption of fresh concrete block pavers. For this purpose, a total of five mixes were designed, including a control mix (No. 1) made with regular tap water, and four mixes that passed through the permanent magnet at a constant speed and water flow of 2.25 m/s 10, 20, 40, and 80 times (mixes No. 2–5), respectively. All of the mixes had the same water-to-cement ratio of 0.46, and the cement, water, and coarse and fine aggregates that were used to prepare 1 m^3^ of the concrete mix was 516 kg, 240 kg, 1150 kg, and 612 kg, respectively. The mix design variable is the number of times that water passed through the permanent magnet (10, 20, 40, and 80).

### 2.3. Specimen Preparation

In order to prepare the concrete mixes, 50 L of regular tap water were first passed through a permanent magnetic field at a constant speed of 2.25 m/s 10, 20, 40, and 80 times. After magnetizing the regular tap water in order to prepare the concrete mixes, the coarse and fine aggregates were first mixed inside the drum mixer. Next, the cement was added and mixed for about two minutes. Then, the water was added and mixed until reaching a homogeneous mixture. Then, the mix was cast in plastic moulds sized 50 mm in height, 100 mm in width, and 200 mm in length. The specimens were demolded after 24 h, cured by immersion in lime-saturated water, and kept at room temperature for 27 more days.

### 2.4. Test Methods

#### 2.4.1. Compressive Strength

The compressive strength of concrete block pavers was determined in accordance with ASTM C140. A load-controlled hydraulic jack with a capacity of 3 MN was used. The load was gradually applied by the hydraulic jack. The specimens were tested 7, 14, and 28 days after the casting date. The mean ultimate compressive stress of at least five specimens was reported as the compressive strength of each mix.

#### 2.4.2. Splitting Tensile Strength

The splitting tensile strength was determined on concrete block pavers according to British Standard (BS) 6717. The tests were carried out along the longest splitting section of the concrete block pavers. Prior to testing, each block specimen was concentrically packed with two steel packing pieces on the top and bottom faces in contact with the plates of the loading machine (Azmoon Saz Mabna, Tehran, Iran). A load-controlled hydraulic jack with a capacity of 3 MN was used. The load was gradually applied by the hydraulic jack until the concrete block paver was split into halves. The mean failure load of at least five specimens was recorded, and the splitting tensile strength was calculated based on the failure load. The specimens were tested 7, 14, and 28 days after the casting date.

#### 2.4.3. Flexural Strength

The flexural strength of concrete block pavers was determined by using a 20-ton capacity device. The specimens were crashed after 7, 14, and 28 days after the casting date. The mean average of five specimens was reported as the ultimate flexural strength of each mix.

#### 2.4.4. Mass Loss

Mass loss is a simple and traditional measuring factor in acidic attack tests [28]. To determine the resistance of concrete block pavers to sulfuric acid attack, a similar preparation method to that of the compressive strength test was used. The specimens were cured by immersion in lime-saturated water and kept at room temperature for 28 days. Afterwards, they were exposed to 5% by weight of an H_2_SO_4_ solution with pH 1.0. The solution was monitored refreshed weekly in order to keep the pH constant for a period of 13 weeks at a temperature of 25 °C. Specimens were removed from the solution weekly, rinsed three times with regular tap water to remove loose reaction products, and left to dry for one hour at room temperature before measuring the mass loss. The mean of five specimens was reported as the mass loss of each mix. The mass loss percentage of each specimen was calculated by the following Equation (1):

Mass loss_t_ (%) = ((M_t_ − M_i_)/M_i_) × 100 (1)

where M_t_ is the mass of the specimens at time t (g), and M_i_ is the initial mass of the specimens before exposure to H_2_SO_4_ solution (g).

#### 2.4.5. Water Absorption

Absorption is usually measured by drying the specimens to constant mass, immersing them in water, and measuring the increase in mass as a percentage of dry mass [29]. The water absorption was determined in five specimens sized 50 mm in height, 100 mm in width, and 200 mm in length, which had been cured in lime-saturated water for 28 days. The mean of five specimens was recorded as the water absorption of each mix.

## 3. Results and Discussion

### 3.1. Effect of Magnetized Water on Compressive Strength

**Figure 5:**
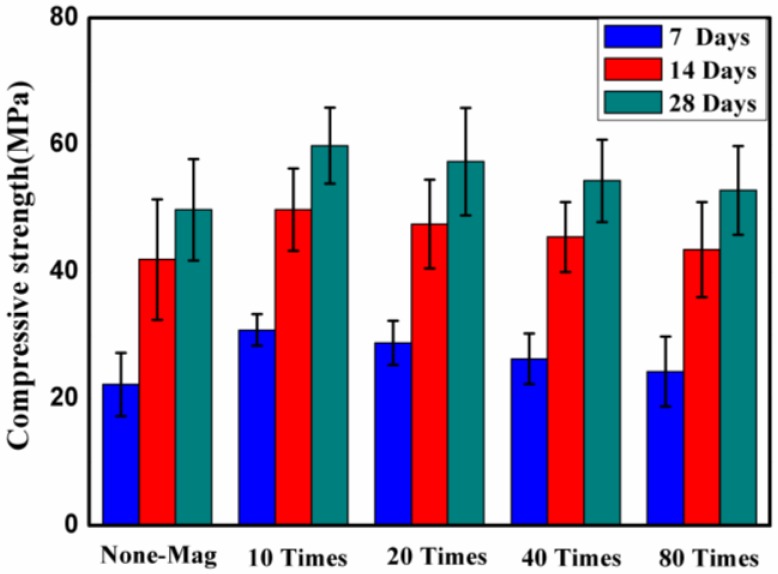
Compressive strength of concrete block pavers after 7, 14, and 28 days of curing in lime-saturated water.

The compressive strength of concrete block pavers with and without magnetized water after 7, 14, and 28 days of curing in lime-saturated water is illustrated in Figure 5. Each point of the plot is a mean value of five independent specimen readings per mix.

As seen in Figure 5, the compressive strength of all of the specimens prepared with magnetized water is higher than that of the specimens prepared with regular tap water at all of the testing ages. This result is in good agreement with previous studies [6,15,16,17,18]. This higher compressive strength may be attributed to the higher specific area of magnetized water relative to regular tap water. As the regular tap water passes through the permanent magnetic field, the size of its clusters and the number of grouped molecules decreases due to the magnetic force, and more water molecules are available for the hydration process. Consequently, more interaction between water molecules and cement particles is expected. This process results in a better quality and density of the hydration products of cement. Therefore, the increase in the hydration of the cement particles may lead to an increase in the compressive strength of the concrete mixes, as mentioned by Ahmed [15]. Figure 5 also shows that the specimens of concrete mix No. 2 displayed the highest increases over time relative to the control mix: about 38%, 21%, and 19% after 7, 14, and 28 days, respectively. The results also showed that there is an inverse relationship between the times that water passes through the permanent magnetic field and the compressive strength of the specimens. In other words, as the number of times that water passes through the permanent magnetic field decreases, the compressive strength of the specimens increases. This may be attributed to the water molecules’ hydrogen bonding. Wang et al. [30] reported that the hydrogen bonding between water molecules is in a dynamic balance after enough magnetized time elapses. When the magnetizing time increases, the balance shifts toward weakening or even breaking the hydrogen bonding in water. Therefore, as the magnetizing time increases, the hydrogen bonding gets weaker, and the friction coefficient becomes lower. Another reason may be the water temperature. As the time that water passes through the water pump increases, a higher temperature is achieved. Therefore, the thermal motion of water molecules is known to become stronger, and thus hydrogen bonding weakens, as mentioned by Jeffrey [31] and Li et al. [32]. The same factors explain the trend on the other mechanical properties, namely the splitting tensile strength and flexural strength.

Figure 5 also shows that, as the curing age grows, the compressive strength of all of the specimens increases, as expected, but the rate of increase varies, as reported previously [18]. The results showed that for specimens prepared with magnetized water, as the number of times that water passes through the permanent magnetic field increases, a higher rate of increase is obtained. In addition, at an early curing age, the compressive strength of specimens with regular tap water increases at a higher rate than that of specimens with magnetized water.

### 3.2. Effect of Magnetized Water on Splitting Tensile Strength

**Figure 6:**
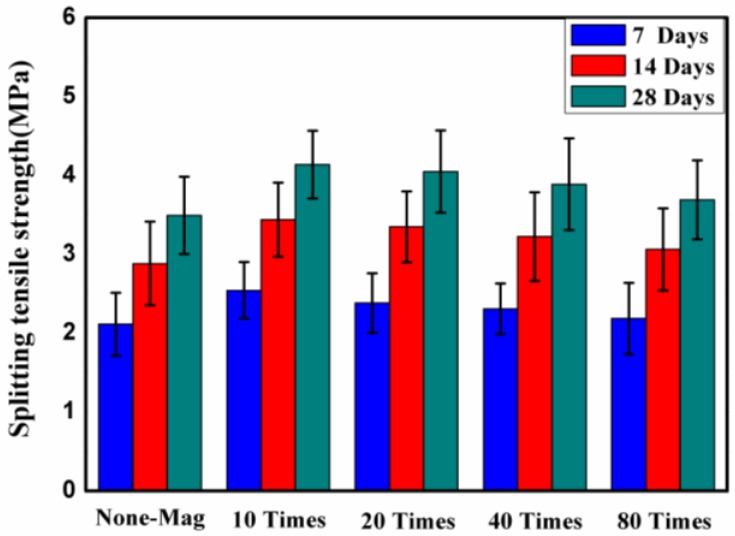
Splitting tensile strength of concrete block pavers after 7, 14, and 28 days of curing in lime-saturated water.

The splitting tensile strength of concrete block pavers modified with magnetized water after 7, 14, and 28 days of curing in lime-saturated water is shown in Figure 6.

Each point of the plot is the mean value of five independent specimen readings per mix. As seen in Figure 6, similar to compressive strength, the splitting tensile strength of all of the specimens with magnetized water at all ages is higher than that of the specimens with regular tap water. This result is in good agreement with previous studies [6,17,19,24]. In other words, magnetized water is more effective than tap water during the hydration process due to the greater activity of the magnetized water molecules. The 28-day splitting tensile strength values for the specimens prepared with magnetized water were approximately between 3.7 and 4.15 MPa, while this value for the control mix was about 3.5 MPa. Figure 6 shows that, similar to compressive strength, the specimens of concrete mix No. 2 displayed the most positive effect of using magnetized water: increases of the splitting tensile strength of around 19% at all ages (after 7, 14, and 28 days of water curing).

It can be concluded that the use of magnetized water instead of tap water can significantly improve the splitting tensile strength of concrete block pavers. Figure 6 also shows that there is an inverse relationship between the splitting tensile strength of specimens with magnetized water and the number of times that the water passes through the permanent magnetic field.

### 3.3. Effect of Magnetized Water on Flexural Strength

**Figure 7:**
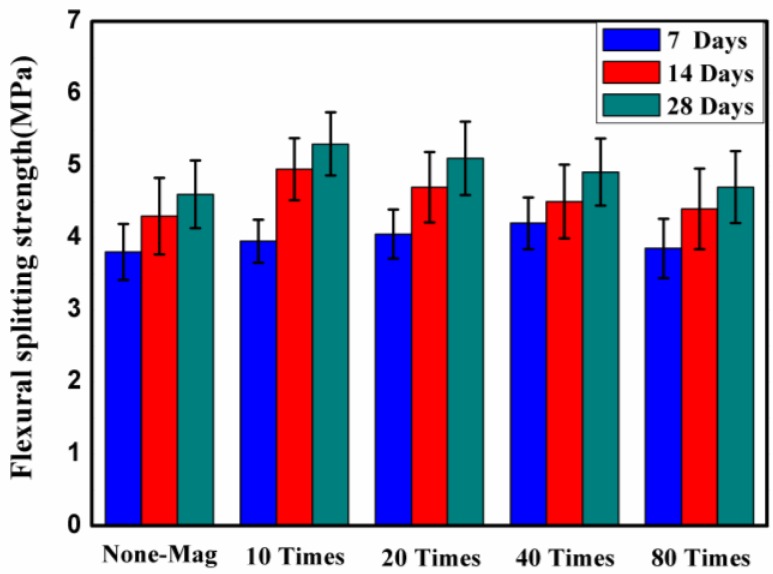
Flexural strength of concrete block pavers after 7, 14, and 28 days of curing in lime-saturated water.

The flexural strength of concrete block pavers cured for 7, 14, and 28 days in lime-saturated water is shown in Figure 7. Each point of the plot is the mean value of five independent specimen readings per mix.

Figure 7 shows that the flexural strength of all of the concrete mixes prepared with magnetized water are higher than that of the control mix: 15%, 11%, 6%, and 3% for specimens with magnetized water that passed through the permanent magnetic field 10, 20, 40, and 80 times, respectively. This result is in good agreement with a previous study [15]. This means that, similar to compressive and splitting tensile strength, passing water through a permanent magnetic field can be an effective way of improving the flexural strength of concrete block pavers. This may attributed to the higher degree of hydration of the specimens with magnetized water. Figure 7 also shows that, similar to compressive and splitting tensile strength, as the number of times that water passes through the permanent magnetic field increases, a lower flexural strength is achieved. Figure 7 also shows that, as the curing age increases, the flexural strength of all of the concrete mixes also increases, as expected, but the rate of increase varies for different concrete mixes. After 14 days of curing, a constant trend can be seen in the flexural strength of concrete mixes with magnetized and regular tap water. The flexural strength of specimens of concrete mix No. 2 displayed the highest increase in flexural strength relative to the control mix: 4%, 15%, and 15.5% after 7, 14, and 28 days of water curing, respectively.

### 3.4. Effects of Sulfuric Acid Immersion

#### 3.4.1. Mass Loss

**Figure 8:**
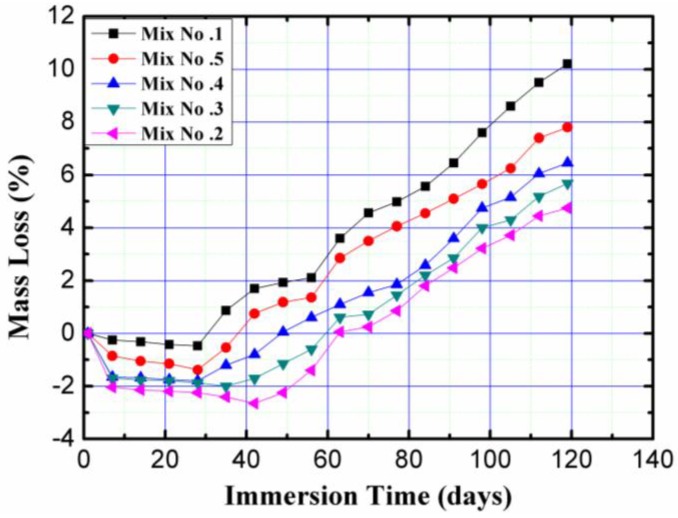
Percentage changes in the mass of the concrete block pavers exposed to 5% by weight of H_2_SO_4_ solution with pH 1.0 versus immersion time.

The percentage changes in the mass of the concrete block pavers exposed to 5% by weight of H_2_SO_4_ solution with pH 1.0 versus immersion time are shown in Figure 8. Each point of the plot is a mean value of five independent specimen readings per mix.

Figure 8 shows that the specimens with regular tap water were more vulnerable to H_2_SO_4_ attack and showed the worst resistance to acid attack compared to specimens with magnetized water, and had a mass loss of 10% after 91 days of exposure. This means that, regardless of the number of times that regular tap water passes through the permanent magnetic field, the magnetized water had a positive and significant effect on the resistance of specimens to acid attack. As seen in Figure 8, for mixes with magnetized water, mix No. 1 displayed the most positive effect from the magnetic field, and had the best resistance to acid attack. Mixes No. 2 to No. 5 displayed 53%, 44%, 37%, and 24% lower mass loss compared to the control mix after 91 days exposure to 5% H_2_SO_4_ solution, respectively. Figure 8 also shows that for mixes with magnetized water, as the number of times that water passes through the permanent magnetic field decreases, the mass loss of the mixes declines gradually. This means that there is an inverse relationship between the number of times that water passes through the permanent magnetic field and the resistance to acid attack. When sulfuric acid reacts with the hydration products, dissolution of the hydrated composites and hydrogen ions occurs [33]. The speed of this action depends on the pore structure, porosity, sulfuric acid concentration, and pH value of the solution [34]. The higher resistance of specimens with magnetized water to acid attack may be attributed to the reduction of pores in the structure of the specimens with magnetized water, as a result of their greater density and higher degree of hydration. This is in good agreement with the results of Ahmed [15], which used magnetized water instead of regular tap water, and found a significant improvement in the microstructural properties of concrete mixes. Consequently, the structure of concrete with magnetized water becomes denser, and lower amounts of pores can be seen in concrete with magnetized water compared to concrete with regular tap water [6,8,15,18]. These differences explain why the magnetized water can increase the durability properties of concrete mixes. The subsequent decrease for more than 10 times the water passing through the permanent magnetic field has been explained in the discussion of the compressive strength results.

#### 3.4.2. Compressive Strength Loss

**Figure 9:**
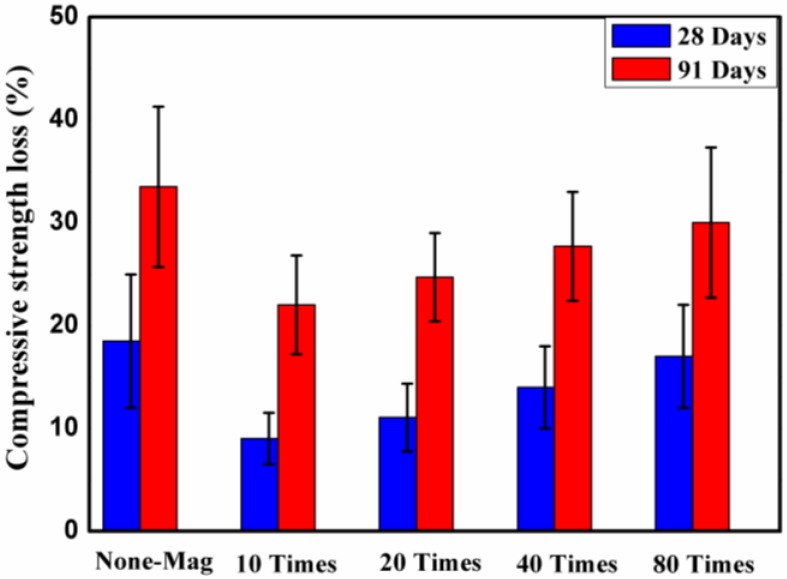
Degradation and percentage changes in the compressive strength of concrete block specimens exposed to 5% by weight of H_2_SO_4_ solution after 28 and 91 days of exposure.

Figure 9 shows the degradation and percentage changes in the compressive strength of concrete block specimens exposed to 5% by weight of H_2_SO_4_ solution after 28 and 91 days of exposure. Each point of the plot is a mean value of five independent specimen readings per mix. The percentage change in the compressive strength of each mix was determined by comparing the compressive strength of specimens after 28 and 91 days of exposure to H_2_SO_4_ solution with the compressive strength of specimens after 28 days of water curing.

Figure 9 shows that the compressive strength of all of the mixes decreased after 28 and 91 days of exposure to H_2_SO_4_ solution, but the rate of decrease varied for different mixes. For all of the concrete mixes, the maximum loss in the compressive strength of concrete mixes was observed after 91 days of exposure to H_2_SO_4_ solution, as expected. The reduction in the compressive strength of the mixes was likely due to dimension decrements and the loss of surface stiffness [35]. The reduction in compressive strength of the mixes may also be attributed to the reaction of sulfuric acid with Ca(OH)_2_ [33]. Allahverdi and Škvara [36] reported that sulfuric acid attack causes the extensive formation of gypsum in the regions close to the surfaces, and tends to cause disintegrating mechanical stresses that ultimately lead to spalling and exposure of the fresh surface. The results also showed that, regardless of the number of times that water passes through the permanent magnetic field, the specimens with magnetized water had a lower loss in compressive strength compared with the ones with regular tap water. This may be attributed to the more compact and dense microstructure of the mixes with magnetized water, which reduced the effective pores in the concrete surface, and hence reduced its permeability. The lower loss in the compressive strength of the specimens with magnetized water may also be related to the lower mass loss of the specimens with magnetized water compared to the ones with regular tap water. The highest loss in compressive strength of the mixes after 91 days of immersion in H_2_SO_4_ solution was registered for mix No. 1. For specimens with magnetized water, the compressive strength percentage loss decreased as the number of times that water passed through the permanent magnetic field reduced. After 91 days of exposure, differences of 11.5%, 9%, 6%, and 3.5% in percentage loss were noted between mix No. 1 and mixes No. 2, No. 3, No. 4, and No. 5, respectively. These results are in agreement with the mass loss results.

### 3.5. Effect of Magnetized Water on Water Absorption

**Figure 10:**
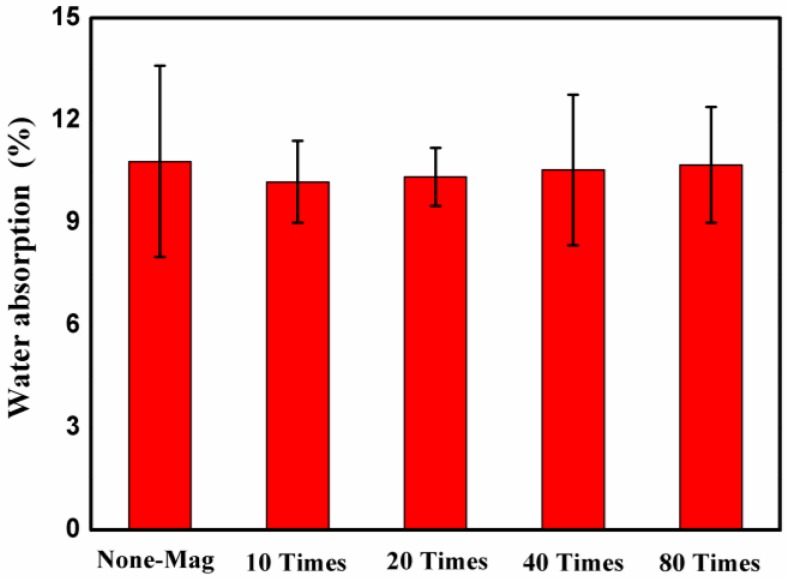
Water absorption of mixes prepared with regular tap water and magnetized water after 28 days of curing.

The water absorption of the mixes prepared with regular tap water and magnetized water after 28 days of curing in lime-saturated water is shown in Figure 10. Each bar of the plot represents the mean value of five independent measurements. As seen in Figure 10, the water absorption of all of the mixes with magnetized water was lower than that of the specimens with regular tap water.

This result is in good agreement with previous studies [17,24], which reported an improvement in the water absorption of concrete specimens with magnetized water. The water absorption of specimens with magnetized water varied between 10.2% and 10.7%, i.e., the effect of magnetized water on the water absorption of concrete block pavers was not very significant. The lower water absorption of mixes with magnetized water may again be attributed to the reduction of pores in the structure of those mixes. As mentioned before, as regular tap water passes through a permanent magnetic field, the activity of its water molecules increases. Consequently, the pore diameter in the structure of these mixes reduces due to the higher activity of magnetized water molecules. The specimens with magnetized water that passes through the permanent magnetic field 10 times (mix No. 2) displayed lower water absorption by 1.5%, 3.5%, and 5% compared to the specimens from mixes No. 3, No. 4, and No. 5, respectively. Mixes No. 2 to No. 5 displayed 6%, 4.5%, 2.5%, and 1% lower water absorption than the control mix after 28 days of curing in lime-saturated water, respectively. Therefore, the magnetized water had a greater effect on the mechanical properties of concrete block pavers than on their water absorption.

### 3.6. Effect of Magnetized Water on Microstructure of Concrete

**Figure 11:**
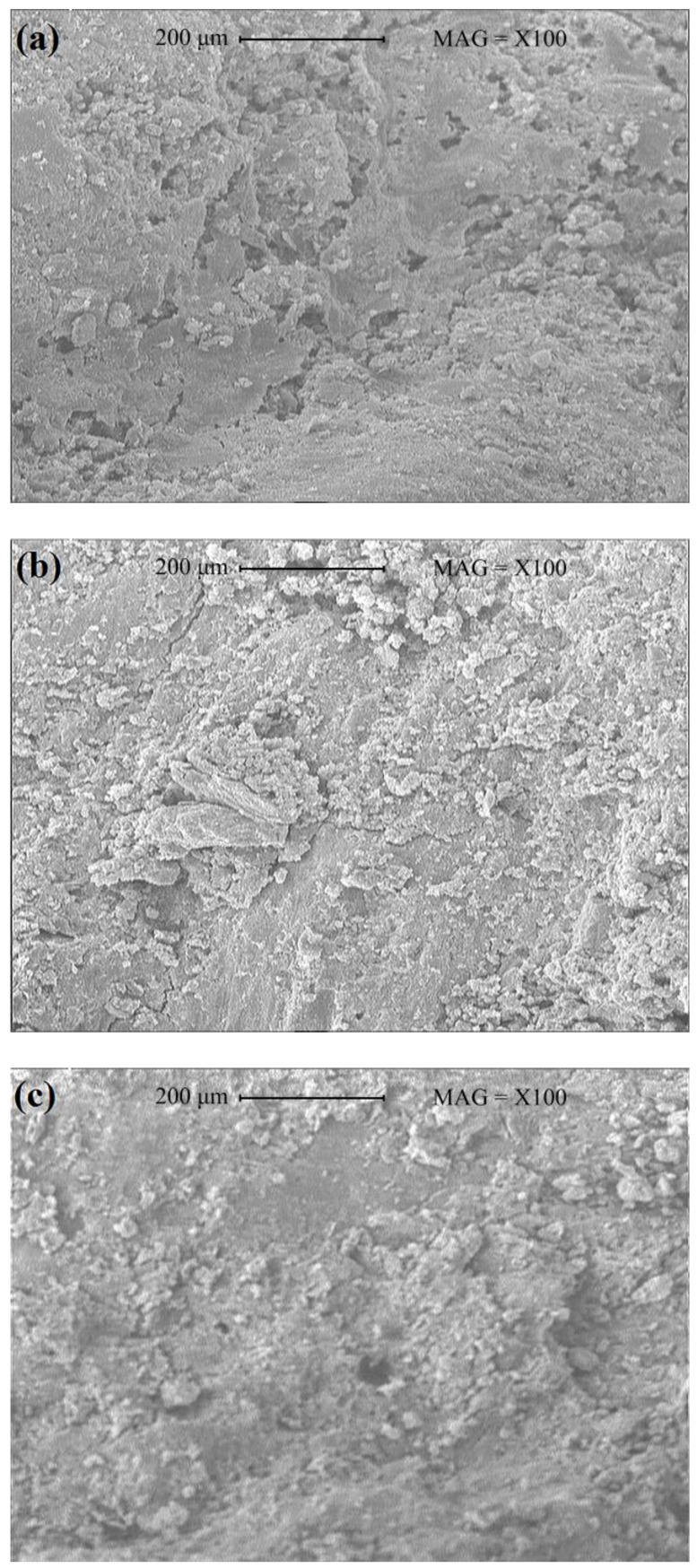
SEM images (100×) of concrete mixes with (**a**) regular tap water; (**b**) magnetized water after passing 10 times; and (**c**) magnetized water after passing 80 times through a permanent magnetic field at a constant speed of 2.25 m/s.

One-hundred magnitude SEM images of concrete mixes with regular tap water and magnetized water that passed 10 and 80 times through a permanent magnet at a constant speed of 2.25 m/s are shown in Figure 11.

**Figure 12:**
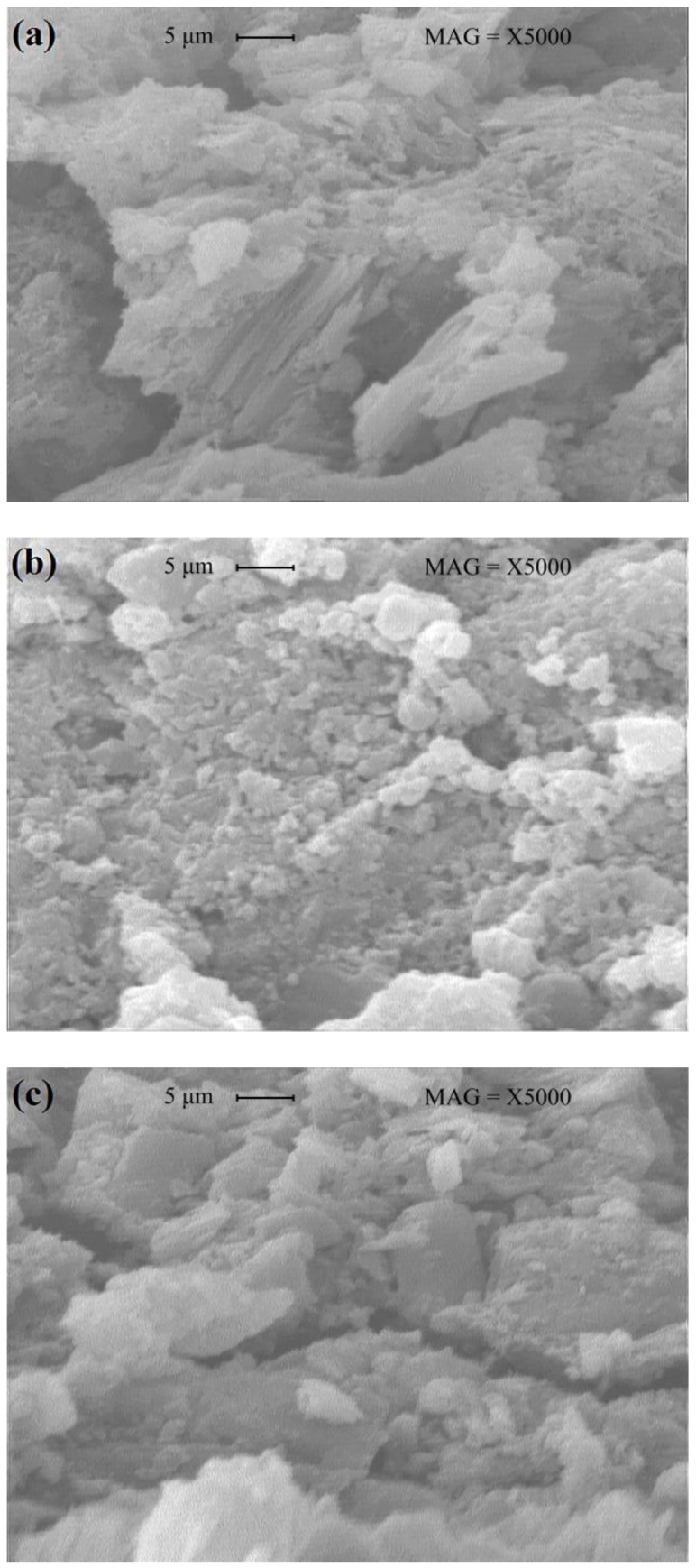
SEM images (5000×) of concrete mixes with (**a**) regular tap water; and (**b**) magnetized water after passing 10 times and (**c**) 80 times through a permanent magnetic field at a constant speed of 2.25 m/s.

The highest amount of pores in the concrete structure occurred in the control mix; i.e., using magnetized water instead of regular tap water led to a significant improvement in the microstructure of the concrete mixes, which agrees with previous studies [6,8,15,16]. This greater density of concrete mixes with magnetized water may be attributed to the higher degree of cement hydration, as a result of more interaction between the cement particles and water molecules. As the number of times that water passes through the permanent magnetic field decreases, the concrete mix becomes denser, and the pores of the concrete structure decrease. Five-thousand magnitude SEM images of the concrete mixes are shown in Figure 12.

Larger and more frequent crystals can be seen in the concrete mixes with magnetized water compared to the control mix. From Figure 11 and Figure 12, it can be concluded that the higher compressive strength, splitting tensile strength, flexural strength, acid attack resistance, and lower water absorption of concrete mixes with magnetized water are due to their higher density and the lower pore content in their structure.

## 4. Conclusions

In this research, the effect of magnetized water on the mechanical and durability properties of concrete block pavers, namely their compressive strength, splitting tensile strength, flexural strength, resistance to sulfuric acid, and water absorption have been investigated, and the following conclusions were drawn:

- The mechanical performance of concrete showed an improvement due to using magnetized water instead of regular tap water: relative to the control mix, an average improvement of 12.5%, 13%, and 9% after 28 days of water curing was registered for the compressive strength, splitting tensile strength, and flexural strength, respectively;
- The results showed that as the curing age increases, the compressive strength, splitting tensile strength, and flexural strength of all of the mixes increases, as expected. However, the rate of increase varies for different mixes;
- The mass and compressive strength loss and water absorption results showed that magnetized water had a positive effect on the resistance to sulfuric attack and water absorption of the concrete mixes. The improvement grew as the number of times that water passed through the permanent magnetic field decreased;
- For the same mix proportions, concrete mixes with magnetized water will have a higher compressive strength, splitting tensile strength, and flexural strength, and a lower mass/compressive strength loss under acid attack and water absorption than control mix specimens, due to their greater density and more efficient degree of cement hydration;
- The SEM images showed that using magnetized water instead of regular tap water led to a significant improvement of the microstructure of the corresponding concrete mixes and resulted in a denser structure compared to the control mix;
- The cost of magnetizing water is very low because of the simple devices used. In this study, the following devices were used, with a total a cost of approximately ($600 USD): (a) an electric pump, (b) two water tanks, and (c) one permanent magnetic field. The cost would have to be adapted to the scale of the work involved. The time needed to pass 10 L of regular tap water through the permanent magnet in this study was about 28 s. This time would decrease as the strength of the electric pump increased.

## Figures and Tables

**Table 1:** Chemical composition of the cement.

Material	Chemical Composition (%)									
	SiO_2_	CaO	Al_2_O_3_	Fe_2_O_3_	MgO	SO_3_	K_2_O	Na_2_O	CL *	LOI **
Cement (Type II)	21.65	63.25	4.3	3.45	2.8	2.05	0.6	0.5	0.07	1.35

* Chlorine. ** Loss on ignition.

**Table 2:** Physical properties of fine and coarse aggregates.

Properties	Fine Aggregates	Coarse Aggregates
Water absorption (%)	4.15	1.63
Existent moisture (%)	3.8	0.51
Modulus of fineness	-	4.79
